# Parasitic Protozoa: Unusual Roles for G-Quadruplexes in Early-Diverging Eukaryotes

**DOI:** 10.3390/molecules24071339

**Published:** 2019-04-05

**Authors:** Franck Dumetz, Catherine J. Merrick

**Affiliations:** Department of Pathology, University of Cambridge, Cambridge CB2 1QP, UK; fd353@cam.ac.uk

**Keywords:** G-quadruplex, G4, protozoa

## Abstract

Guanine-quadruplex (G4) motifs, at both the DNA and RNA levels, have assumed an important place in our understanding of the biology of eukaryotes, bacteria and viruses. However, it is generally little known that their very first description, as well as the foundational work on G4s, was performed on protozoans: unicellular life forms that are often parasitic. In this review, we provide a historical perspective on the discovery of G4s, intertwined with their biological significance across the protozoan kingdom. This is a history in three parts: first, a period of discovery including the first characterisation of a G4 motif at the DNA level in ciliates (environmental protozoa); second, a period less dense in publications concerning protozoa, during which DNA G4s were discovered in both humans and viruses; and third, a period of renewed interest in protozoa, including more mechanistic work in ciliates but also in pathogenic protozoa. This last period has opened an exciting prospect of finding new anti-parasitic drugs to interfere with parasite biology, thus adding new compounds to the therapeutic arsenal.

## 1. Introduction

Traditionally DNA is viewed by the public, and indeed by the vast majority of scientists, as a double helical structure composed of four nucleotides, pairing adenine-thymine (A-T) and cytosine-guanine (C-G) [1], as observed by Watson and Crick in 1953. However, numerous publications have now demonstrated that DNA can be arranged to form different secondary structures, including secondary motifs based on non-Watson and Crick pairing. Such pairing is notable in two motifs, the guanine-quadruplex (G4) and the i-motif composed of cytosine residues [2,3]. These structures can form when the DNA is single stranded, thus primarily during replication, and potentially also during transcription [4]. 

**Figure 1:**
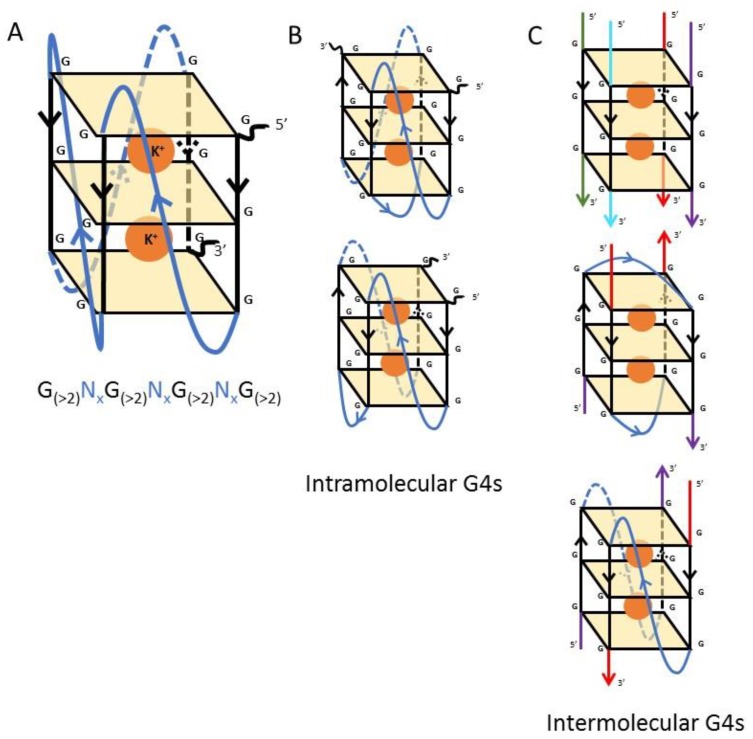
Schematic representations of G-quadruplex structures. (**A**) Schematic representation of a parallel G-quadruplex: the three planar G-stacks are represented in yellow, the T-loops or polypurine tracks are represented in blue and stabilising potassium cations, in orange. (**B**) Intramolecular G4s in two antiparallel configurations. (**C**) Intermolecular G4s in both parallel and antiparallel configurations: structures involving four DNA molecules (red, purple, blue and green) or two DNA molecules (red and purple) are shown.

**Figure 2:**
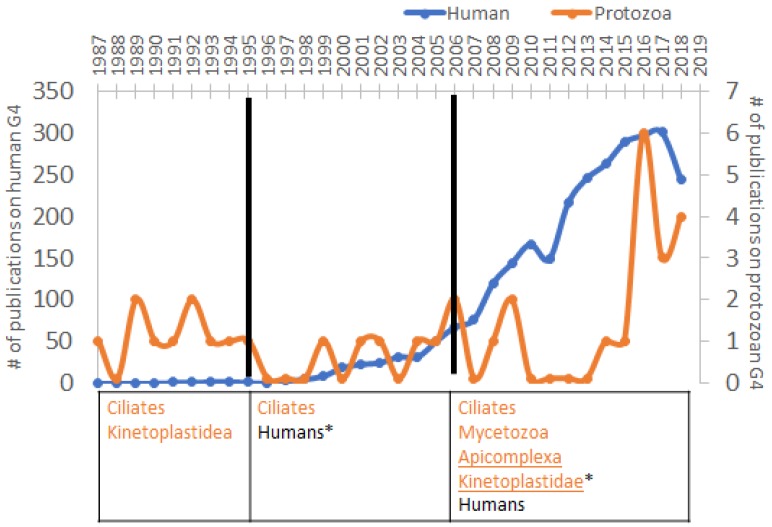
Compared evolution of the number of G4 publications related to protozoa and humans across time. Data were extracted from PubMed on 20/02/19 using the search terms “G-quadruplex human”, “G4 humans” and “Guanine quadruplex humans”, then compared to eliminate duplicates. The protozoan literature count is derived from the publications reviewed here, which represent the totality of the work available so far in the field. Orange line and taxa represent the protozoan data and the blue ones represent the human data. Underlined taxa include studies of human pathogens. * represents clades where rG4 work is available.

The G4 motif is a family of structurally-diverse four-stranded secondary structures composed of stacks of planar guanine tetrads connected by intervening loop sequences following the general sequence G_(>2)_N_x_G_(>2)_N_x_G_(>2)_N_x_G_(>2)_ [5] (Figure 1). This motif is stabilised in the presence of K^+^, or to a lesser extent Na^+^, and destabilised in the presence of Li^+^ [6]. In the human genome, G4s are enriched in key regulatory sites, including oncogene promoters and telomeres, as well as appearing more generally in gene sequences and 5′UTRs [7]. Studies carried out on the functionality of G4 motifs have implicated them in oncogenesis, telomere structure and gene expression regulation [8]. A previous review from our group summarised the implications of G4s for the biology of many micro-organisms, mainly viruses and bacteria as well as a few protozoans of clinical interest. It highlighted a strong role for these motifs in immune evasion and virulence [9]. In this present review we focus first on the historical perspective brought by the fact that G4s were originally characterised in environmental protozoans. Then, after the first demonstration that G4s were also present in human cells, the field shifted towards research on human cells and viruses. It was only in the mid-2000s that G4 research again took an interest in protozoans and began to examine their biological significance, mostly with the idea to generate new anti-parasitic drugs (Figure 2).

## 2. To Begin at the Beginning: A Free-Living Protozoan

Protozoans are ancient forms of eukaryotic life, now considered as early-diverging from more “classical” eukaryotes. However, the original observation of the different forms of G4s, as well as their further characterisation, was made on environmental protozoans of the Ciliophora phylum, like *Oxytricha* and *Tetrahymena.* This was due to two genomic particularities in these organisms: (1) the multiplicity of their genomic fragments (~16,000 different molecules of DNA for the former [10] and more than 200 for the latter [11]), and (2) the presence of a highly guanine-rich telomeric repeat, T_4_G_4_, on each fragment [12]. These two features rendered the investigation of GC-rich sequences much easier. In 1987, Oka et al. demonstrated the DNA/DNA interaction of the telomeric guanine clusters using macromolecular genomic DNA from *Oxytricha nova.* They hypothesised three different spatial conformations for this arrangement: antiparallel triplex, antiparallel quadruplex and parallel quadruplex [6]. It was in 1989 that Williamson et al. proposed clearly “the G-quartet model” based on their investigation of telomeric shape in *Oxytricha* and *Tetrahymena.* Using synthetic oligos of the telomere sequence in various salt conditions they demonstrated that in the presence of K^+^, Na^+^ and Cs^+^, telomeres had an increase in electrophoretic mobility compared to telomeric sequences in presence of Li^+^ or no salt [13]. Further characterisation of the G4 motif was pursued using dimethylsulfate (DMS), a chemical that methylates guanine residues on N7 or O6. Thus, the G-G bonding in position N7 was characterised and the first model of an anti-parallel G4 was proposed, using two telomeric sequences of *Tetrahymena* with four stacks of guanine [14].

At this time a fraction of the research turned to investigate the biological function of G4s in telomeric sequences. Investigating the kinetics of dissociation of K^+^- and Na^+^-stabilised G4s, Raghuraman et al. defined for the first time the period required to unfold these structures at 37 °C. The G4s had a lengthy half-life of 4 h for the Na^+^-stabilised and 18 h for the K^+^-stabilised version, suggesting that there might be an active, protein-mediated unfolding mechanism in vivo. They also compared the ability of *Oxytricha* telomere-binding-protein to bind to three different states of DNA: fully-folded G4, intermediate G4 and non-folded DNA. Protein-binding was not observed in the fully-folded structure, whereas the two latter structures were a good substrate for *Oxytricha*’s telomere-binding protein [15]. Finally, K^+^ was also confirmed to be the most stabilising monovalent cation via biochemical rather than biophysical methods [16]. All this information implied the likelihood of G4 folding in cellulo, since potassium is the most abundant cation inside the cell. It also highlighted the high stability of the G4 structure and the need for an active unfolding mechanism allowing the access of DNA binding proteins.

After the discovery and primary characterisation of G4 formation, the next step, from 1992 to 1994, was to characterise the G4 at the atomic level. Several teams then either used crystallography or nucleic magnetic resonance spectroscopy of the telomeric G4 from *Oxytricha*. From a modern perspective, all this work could theoretically have been conducted on any G4-forming oligonucleotide, but since G4s were primarily recognised at this time in ciliate telomere sequences, these remained the model system of choice. First, using K^+^ as the stabilising cation, the spatial organisation of the arrangement between the different guanine residues of the quadruplex was characterised, showing that both *syn-* and *anti-* bonding of the different guanine residues is possible [17]. This work was further supported and deepened by the additional structures of two different forms of G4, the intermolecular G4, and the intramolecular G4 (in which a single DNA strand encoding a canonical G4 sequence forms the secondary structure within itself) [13]. At the same time, four different topologies of the G4 were described, all depending on the charge, and thus the sequence, of the so-called T-loop [18,19] (Figure 1). Later work would show that the type of conformation is also defined by the length of the T-loop [20]. The work of Schultze et al. also paved the way to the structural description of parallel and antiparallel G4s. Firstly, two different conformations were described for the G4 formed from two single-stranded DNA molecules: (1) the two molecules are oriented in the same direction (5′ to 3′// 5′ to 3′), thus forming a parallel conformation, and (2) the two molecules are oriented in the opposite direction (5′ to 3′// 3′ to 5′) making an anti-parallel conformation [18]. Concomitantly, Lu et al. in 1993 unravelled the difference in thermodynamics between parallel and anti-parallel G4s, measuring a higher stability of the parallel G4 in the presence of Na^+^ [21].

Finally, this flourishing period for G4 discovery and characterisation was brought to a close with a publication on a so-far unexplored protozoan from the class of *Kinetoplastida*—the plant pathogen *Phytomonas serpens*, a Trypanosomatid. *P. serpens* has a nucleus and also a kinetoplast, which is a *Kinetoplastidea*-specific organelle functioning like a mitochondrion and containing the kDNA. The kDNA is a made up of two different families of circles, the maxi- and the mini-circles [22]. Based on oligonucleotide sequences of the minicircles exposed to DMS in G4-forming conditions, the authors described the potential for parts of the minicircles to form intramolecular G4s [23]. This was the first description of G4s in a protozoan outside of the *Ciliophora* family and it would be another 20 years before G4s were further investigated in *Kinetoplastid* parasites, including those that are important human pathogens.

## 3. Increased Interest in G4s in Humans Correlates with the Diminution of Attention to Protozoa

For more than ten years (1995 to 2006), only a few papers were published on G4s in protozoans. However, after the first period of description and characterisation, a series of articles began to appear related to the biological relevance of G4s, and some of these again took advantage of ciliate protozoa. First, some G4 structure-specific antibodies were produced: an important new research tool for the field. Using another *Ciliophora* telomeric sequence, that of *Stylonychia lemnae*, Schaffitzel et al. produced two different antibodies using ribosome display: Sty3 that binds to parallel and antiparallel G4s and Sty49 with higher specificity for antiparallel G4s. Two very interesting biological observations were made: (1) there is no trace of staining of the replication band, indicating that, in ciliates, G4s are resolved during replication, and (2) that G4 motifs were only present in the macronucleus of *Stylonychia* and not in the micronucleus, suggesting a difference of DNA organisation between the two nuclei [24]. This observation highlights a very unusual feature of these ciliates: *Stylonychia* has two nuclei assuring two different biological functions. The macronucleus is the centre of the metabolic activity and undergoes drastic remodelling during the reproductive life cycle, while the micronucleus codes for the germline genetic material [25]. It is notable, however, that although Sty49 was the first antibody to detect G4 motifs, it was not used subsequently to study G4s outside of ciliates. This fact has some potential explanations: the number of telomeres in ciliates creates a very high density of G4s, and the G4 sequence of the ciliate telomeres is more stable than the human one, having one more stack of guanine. It was only in 2013 that Biffi et al. produced an antibody, BG4, binding to human G4 motifs [2].

A few years later, using the Sty49 antibody, Paeschke et al. published the first functional study on the regulatory role of telomeric G4s in *S. lemnae*. They reported the essentiality of the two Telomere End Binding Proteins, TEBPa and TEBPb, for the formation of G4s in vitro and in vivo, and further demonstrated that TEBPb phosphorylation was necessary for G4 formation [26]. Meanwhile, working on the interaction between telomere-associated proteins and G4 motifs, Oganesian et al. focused their investigation on telomerase activity in two ciliates, *Tetrahymena* and *Euplotes*. In a primer extension assay using Na^+^-stabilised intermolecular G4s and telomerase from both organisms, they described the intermolecular G4 as being an excellent substrate for the enzyme, suggesting that, at least in vitro, telomere extension is mediated by the presence of the G4 motifs [27].

Secondly during this period, more structural work was performed using NMR and crystallography on *Oxytricha* and *Tetrahymena* sequences, changing the stabilising cations in G4s in crystal form or in solution [28,29,30,31]. The first crystal of a G4 structure from a protozoan interacting with a G4-binding protein was reported: Horvath and Schultz described the interaction between *Oxytricha nova* TEBP and Na^+^-stabilised G4 telomeric sequence [32]. Thus, as widespread interest in the G4s in human cells, and particularly in cancer cells, began its exponential growth, some important work did continue to use protozoa as tractable model systems for the study of G4s.

## 4. Biological Significance of G4s in Environmental and Pathogenic Protozoans

The third period of the history between G4s and protozoa saw the research expand to pathogenic protozoa as well. This period can be split into two different paths. First, the characterisation of the motif and its various biological functions was further pursued in ciliates and then extended to eukaryotic pathogens. Second, G4s began to be pursued as a potential target for anti-parasitic drugs.

### 4.1. G4 Structures and Functions in Protozoa

This period opened with the discovery of a previously-undescribed form of G4: the V4-motif introduced by Nielsen et al. To achieve this, they used the *Oxytrichia* T_4_G_4_ telomere repeat with the addition of locked nucleic acids, which are analogues of nucleic acids with a locked ribose in C3′-*endo*, forbidding a *syn* or *anti* conformation depending on the pre-existing chemical arrangement [33]. The authors speculated that V4 structures could form in certain conditions; however, it is yet to be demonstrated that this fold is actually physiological, either in *Oxytrichia* or elsewhere. In fact, the G4 research community remains preoccupied to this day with the question of whether all motifs observed in vitro can actually exist in vivo as well.

Secondly, the importance of sequence diversity within the G4 motif itself, which had thus far been largely neglected, was now studied, with ciliate telomeres again being the model sequences of choice. For this work, the highly homogenous T_4_G_4_ repeat was clearly an ideal model. First, Abu-Ghazalah et al. in 2009 looked at the composition of the G-track and the T-loop of *Oxytricha* G4s. Using oligonucleotide sequences of *Oxytricha* telomere repeats with the addition or deletion of a G in the G-track or a T in the T-loop, they showed that stability correlates with the number of guanine residues. They then demonstrated an effect of the T-loop length on the global conformation. Lastly, they ran stability experiments with all residues of the T-loop replaced by adenosine, showing different behaviours of G4s with T-loops versus A-loops [20]. Enforcing the first statement that stability increases when more guanines are present in the motif, Demkovičová et al. “humanised” the G4 sequence of *Tetrahymena* by changing the first guanine of the G-track to an adenine (T_2_G_4_ -> T_2_AG_3_). They showed that the substitution does not impair G4 formation but may change the topology of the G4 and also reduce its stability [34]. This work highlights an important transition away from studies of highly homogenous ciliate telomere sequences (which were originally chosen for their very homogeneity), towards studies of a greater diversity of G4-forming sequences.

Indeed, the second conclusion of the work done by Abu-Ghazalah et al. opened the perspective to study diverse, non-telomeric G4 motifs, which also occur in ciliate genomes. Ciliates like *Oxytricha* and *Tetrahymena* restructure their somatic genome during sexual differentiation and during this process up to a third of the genetic material is excised and lost. The lost fragments, also called internal eliminated sequences (IES), have the particularity to be outside of the telomeric regions and flanked by cis-acting sequences, one of the most studied being the polypurine tract (A_5_G_5_) [25,35,36]. While confirming that A_5_G_5_ is a G4 forming sequence, Carle et al. demonstrated that the protein Lia3 was binding to parallel G4s, as well as ensuring DNA cleavage. When the *LIA3* gene was knocked out, excision of the IES was prevented and the presence of the G4 was also essential for the binding of Lia3 [37]. Thus, G4s in ciliates evidently play specific biological roles beyond those in telomere maintenance. Over the past decade, as work on G4s has extended to pathogenic protozoa which also have unusual basic biology, it has become increasingly clear that this is a common theme: G4s can play interesting species-specific roles in the biology of many different protozoan parasites.

### 4.2. G4 Motifs in an AT-Rich Genome: The Example of Plasmodium Falciparum

Using bioinformatic tools, it was reported in 2007 that 40% of human gene promoters are carrying a Putative G-Quadruplex Sequence (PQS), and that PQSs appear on average approximately every kilobase in this genome [38]. This, therefore, is the PQS distribution that might be ‘expected’ in similarly G-C/A-T balanced genomes. However, not all eukaryotes have such genomes and nucleotide imbalance is a particularly striking feature of some protozoan parasites: certain malaria parasites, for example, have A-T content exceeding 80%, while *Leishmania* parasites are G-C-biased.

In 2009, Smargiasso et al. published the first bioinformatic PQS screen of the genome of the malaria parasite *Plasmodium falciparum*, which is extremely A-T-biased. They identified only 63 PQSs in non-telomeric sequences and, interestingly, these were concentrated in the upstream regions of a subset of the *var* virulence genes (the ‘group B’ *var* genes). This gene family encodes the major variant antigen of this parasite, *P. falciparum* Erythrocyte Membrane Protein 1 (PfEMP1), which is expressed at the surface of the infected erythrocytes. Each parasite genome has ~60 *var* variants; their expression is mutually exclusive and is regulated by epigenetic silencing and switching [39]. The authors demonstrated that G4s can indeed form within the *var* B upstream sequence, leading to a new hypothesis that *var* gene expression might be regulated via structure-specific helicases that can target G4s [40]. Two recent papers have now addressed this idea by knocking out one of the two RecQ helicase homologs found in *P. falciparum,* called *Pf*BLM [41,42]. Both papers reported changes in *var* gene expression, but curiously Claessens et al. found that *var* gene expression increased in their knockout, whereas Li et al. found that expression of the gene family was completely shut down. The reason for this difference remains, at present, unclear.

In order for *P. falciparum* parasites to continually evade the immune system, antigenic switching amongst a ~60-gene family is not enough: *var* genes must also recombine to create new variants [43]. In 2016, Stanton et al. implicated G4s in this process of recombination, in addition to the regulation of *var* gene expression. Stanton et al. found 80 PQSs in an updated version of the *P. falciparum* reference genome and reported a strong spatial association between PQSs and *var* gene recombination events. Furthermore, it was again suggested that G4-targetting DNA helicases could regulate this process in the parasite [44]. Indeed, the same group subsequently demonstrated the importance of a *P. falciparum* RecQ helicase in *var* gene recombination. This time, the *Pf*WRN helicase was knocked down, and recombination events in the *var* gene family dramatically increased (whereas knockout of the parasite’s only other RecQ homolog, *Pf*BLM, had no such effect) [41]. Overall, the *P. falciparum* parasite has evidently evolved to retain G4-forming sequences in a highly A-T-rich genome for a very specific purpose: regulating both the expression and the evolution of a key virulence gene family.

Finally, in 2016, Bhartiya et al. performed another bioinformatic G4 screening on 6 different species of *Plasmodium*. They included 2-quartet as well as 3-quartet motifs and therefore described a higher number of PQSs (conventionally, three quartets are considered necessary for a stable DNA G4, but two may be sufficient for an RNA G4 (rG4)). Concentrating on these potential rG4s, they then re-analysed various RNA-seq datasets and ribosome footprinting studies and concluded that translation efficiency was reduced at certain lifecycle stages in PQS-harbouring genes [45]. A mechanistic basis for this observation has yet to emerge, but this was the first report to focus on rG4s in *P. falciparum*, and the first to suggest a G4-based expression-regulating system in a protozoan.

*P. falciparum* has the most A-T-rich genome yet sequenced, but other protozoa do exist with similarly biased genomes. One such organism is the environmental Mycetozoa *Distyostelium discoideum*. Bedrat et al. recently developed a bioinformatic program, G4Hunter, which uses a new and more sophisticated algorithm to detect the presence of G4 motifs based on genome sequences: they included two A-T-rich protozoa in their analysis, *D. discoideum* and *P. falciparum*. The number of predicted G4s from this new algorithm remained low, depending on the parameters: between 249 and 1055 for *D. discoideum* (and even lower for *P. falciparum*), but the authors hypothesised that the few PQSs retained in the *D. discoideum* genome could be informative to study [7]. Therefore, the same team solved the crystal structure of a non-telomeric G4-forming sequence (A_5_G_5_) located in a putative promoter of two divergent *D. discoideum* genes [46]. A biological function for this G4 has yet to be established, but this last publication at least physically confirmed the presence of G4 motifs outside of non-telomeric regions in this protozoan.

### 4.3. G4 Motifs in Transcription Control and RNA Editing in Trypanosomatids

After the demonstration of G4 formation in *P. serpens* kDNA [23], the field remained unexplored in the *Trypanosomatid* family. However, interest was raised again in *Trypanosomatidae* with the study of human pathogens such as *Trypanosoma brucei*, two subspecies of which cause human sleeping sickness; *Trypanosoma cruzi*, causing Chagas disease; and *Leishmania spp*, responsible for the complex of diseases called leishmaniasis [47]. It was only in 2015 that Genest et al. performed the first SMRT sequencing of *L. tarentolae*, revealing DNA sequence together with epigenetic base modifications. They demonstrated the presence of PQSs surrounding regions of β-d-glucosyl-hydroxymethyluracil, or ‘base J’, a *Trypanosomatid*-specific epigenetic modification of thymine. The *Trypanosomatidae* group of protozoa is characterised by unusual poly-cistronic transcription: base J is a marker of transcriptional strand-switching between poly-cistrons and it is also present in abundance in the telomeres (99%). They verified their discovery using a plasmid carrying the detected PQS, (GGGTTA)_10_, and found that even plasmids carrying only this sequence without any coding sequence were modified to harbour base J. This result suggests that the presence of a G4 is a hallmark for base J positioning [48]. Again, it implies that G4s have evolved to play a highly specific role in this group of organisms relating to their unique biology – in this instance, their use of polycistronic transcription and base J.

Regarding the same family of parasites, and after the discovery of G4 formation at the RNA level in humans [49], Leeder et al., in a series of two articles, paved the road for rG4 studies in kinetoplastids. Kinetoplastids, as previously stated, carry a kinetoplast that contains the mitochondrial DNA. The RNA molecules transcribed from the kDNA are called kRNA and undergo a unique, complex and drastic editing process (uracil insertion/deletion). This mechanism is driven by a mitochondrial multienzyme complex, the editosome, guided by guide RNA encrypted on the minicircles. The editosome edits the pre-kRNA molecule where the gRNA binds [50]. In 2015, it was demonstrated, using selective 2′-hydroxyl acylation primer extension (SHAPE) on kRNA in different stages of maturation, that pre-kRNA molecules can form several rG4s (up to four rG4s in certain pre-kRNA). In the presence of the editosome, up to fifty percent of the rG4 structures unwound, favouring the formation of pre-kRNA:gRNA hybrid RNAs for maturation into fully functional kRNA [51]. In the second article, where they included the in silico structure of the kRNA of two other species, *T. cruzi* and *L. tarentolae*, they made the same conclusion as previously, and finally hypothesised that kDNA replication versus kDNA transcription is directly linked to the presence of intra-molecular G4 structures in the pre-kRNA. When the rG4 is formed on the neo-synthetised pre-kRNA, it cannot recognise PQSs on the maxicircle, leading to active transcription and inactive maxicircle replication. On the contrary, when the rG4 in the pre-kRNA molecule is unwound, it allows the formation of a kDNA/kRNA hybrid, preventing transcription and activating kDNA replication [52]. Yet again, G4s are evidently being used in sophisticated ways to modulate a highly specific aspect of *Trypanosomatidae* biology.

Overall, G4 motifs have been shown to play several different roles in the biology of protozoa. They either organise the genome, as in ciliates with germline formation or in *P. falciparum* with *var* gene recombination, or they regulate gene expression, as in Trypanosomatids with the placement of base J. Furthermore, the first demonstration of G4 presence at the RNA level—now reported in *T. brucei*—gives a role to the rG4 in regulating transcript editing. Regarding the pathogenic species, most of these mechanisms are essential for parasite survival and this makes G4s potential targets for anti-parasitic drugs.

### 4.4. Targeting the G4 Motifs In Pathogenic Protozoan: A New Strategy in the Design of Anti-Parasitic Drugs

The second path of G4-related research that remains very active today is represented by the discovery of new anti-protozoan drugs to fight pathogenic species. In the era of drug-resistant pathogens, new pharmaceutical compounds are necessary to complete the pharmacopeia. In this regard, non-pathogenic protozoa can be useful as well – before directly working on pathogenic organisms, which require a specialised biosafety level of confinement, ciliates have sometimes been used as models to test anti-G4 drugs [53]. In pathogenic protozoa, meanwhile, there are exciting prospects for the development of G4-targetting drugs, some of which are described below, or alternatively, G4-based aptamers [54], which are as yet little-explored for protozoan parasites but are fast becoming an established research avenue for anti-virals [55].

The very first report of compounds binding to G4s in *P. falciparum* telomeres was made in 2008 by De Cian et al., at the same time as the description of the structure and characteristics of *P. falciparum* G4s. They showed that known G4-binding compounds, developed primarily for human G4s, also bound to *P. falciparum* G4s more effectively than to duplex DNA. However, none of the 12 compounds tested showed a particular discrimination between *Plasmodium* and human G4 structures in vitro [56]. Nevertheless, as for many good drugs, pathogen-over-host specificity isn’t always required, particularly if the pathogen replicates faster than the host cell. Therefore, Calvo et al. tested the telomerase activity and parasite growth of *P. falciparum* when exposed to two drugs previously tested by De Cian et al., TMPyP4 and telomestatin. While the first showed the highest reduction in telomerase activity, the second showed the highest parasite growth inhibition. It is important to note that G4-targetting was not proven and telomestatin activity could interfere with other pathways inside the parasite [57]. However, three other G4-binding compounds were subsequently tested by another research group, and showed significant *P. falciparum* telomere erosion, suggesting a common mode of interference with telomerase activity [58].

While the first compound screens focused entirely on molecules capable of inhibiting telomerase activity, others focused on molecules capable of interfering with the G4s scattered across the genome and regulating different biological functions, as described earlier in this review. Belmonte-Reche et al. recently published a screen of seven carboxy-NDI derivatives against *T. b. brucei*, *L. major* and *P. falciparum*. Amongst the seven, two derivatives showed a particular action against *T. b. brucei* and a limited toxicity in human cells, making them potential drug candidates against the *T. brucei* subspecies responsible for sleeping sickness [59]. Focusing on *P. falciparum*, Harris et al. demonstrated G4-related anti-parasitic activity of a candidate anticancer drug, a fluoroquinolone called quarfloxin. Quarfloxin showed rapid killing activity against the erythrocytic forms of the malaria parasite in culture and could represent a good drug for repurposing against malaria. Unlike telomestatin, quarfloxin did not apparently cause telomere erosion: it may instead exert its toxicity via genes encoding non-telomeric G4s [60].

## 5. Conclusions

As can be observed throughout this review, the history of G4 discovery is intertwined with protozoan biology, from the understanding of genome organisation to drug development. Looking at the large diversity of biological systems that represent the protozoan phylum, it is safe to assume that we still have a lot to learn from these small but far-from-simple organisms. Furthermore, the presence of G4s within the telomeres, regulatory elements and coding regions of many pathogenic protozoa makes them a structure of choice to develop new compounds or repurpose or modify existing ones, thus opening new perspectives for the treatment of major parasitic diseases.
